# Breakfast Eating Habits and Lifestyle Behaviors among Saudi Primary School Children Attending Public Versus Private Schools

**DOI:** 10.3390/children8020134

**Published:** 2021-02-11

**Authors:** Laura Jabri, Amani A. Al-Rasheedi, Rayan A. Alsulaimani, Hazzaa M. Al-Hazzaa

**Affiliations:** 1 American International School of Jeddah, Jeddah 21352, Saudi Arabia; laurajabri@gmail.com; 2 Food and Nutrition Department, Faculty of Human Sciences and Design, King Abdul Aziz University, Jeddah 42751, Saudi Arabia; aalrasheedi@kau.edu.sa; 3 Department of Pharmacology, Faculty of Medicine, King Abdul Aziz University, Jeddah 42751, Saudi Arabia; raalsulaimani@kau.edu.sa; 4 Lifestyle and Health Research Center, Health Sciences Research Center, Princess Nourah bint Abdulrahman University, Riyadh 11671, Saudi Arabia

**Keywords:** breakfast intake, children, lifestyle behaviors, BMI, socio-demographic factors, private schools

## Abstract

We investigated breakfast eating habits and lifestyle behaviors among Saudi school children attending public versus private schools. A random sample of 1149 children (girls: 54.4%) from public and private schools was selected from elementary schools using the multistage stratified cluster method. Measurements included body weight, height, body mass index (BMI), and self-reported questionnaires filled by the child’s parents. There was no significant (*p* = 0.44) difference in the prevalence of breakfast intake between children attending public (20.6%) versus private (19.4%) schools. However, there was a gender by school type interactions in breakfast intake frequency, as boys in private but not in public schools had significantly (*p* = 0.006) higher (26.3%) daily breakfast intake than girls (13.3%). Over 56% of the children ate and drank from the school canteen, and impacting factors on children’s choices were children’s desire, food taste, and parental influence. More parents of children in private (12.1%) than in public (6.9%) schools were satisfied with the food in the school canteen. Younger age (aOR = 0.889, 95% CI = 0.815–0.970, *p* = 0.008), higher father education (aOR = 1.380, 95% CI = 1.130–1.686, *p* = 0.002), family income (aOR = 1.227, 95% CI = 1.005–1.498, *p* = 0.044), and insufficient sleep duration (aOR = 0.740, 95% CI = 0.553–0.990, *p* = 0.042) were significantly associated with being in a private school. Furthermore, no significant differences, when adjusted for socio-demographic factors, appeared in breakfast intake or overweight/obesity relative to school type. Interventions to improve daily breakfast consumption and lifestyle behaviors of Saudi children are warranted.

## 1. Introduction

Habitual breakfast consumption contributes substantially to a child’s physical health and wellbeing by improving nutrient intake and overall dietary quality [1,2,3,4]. A more balanced macronutrient distribution including higher fiber and lower saturated fat intake was found among Canadian children and adolescents who were breakfast consumers [5]. Higher levels of essential micronutrients including vitamin D, vitamin C, and folate have also been observed in European adolescents who consumed breakfast compared to those who skipped the breakfast meal [6]. Conversely, breakfast skipping is associated with several negative health effects and worse anthropometrics including higher body mass index (BMI) z-scores, waist circumferences, and overweight/obesity prevalence among children and adolescents [2,7,8]. A systematic review of studies from 33 countries found that skipping breakfast also negatively altered blood lipid profiles and may be associated with an increased risk for developing the metabolic syndrome, a finding possibly explained by higher postprandial glycemic response in breakfast skippers [9]. Breakfast skipping was also shown to be associated with various suboptimal lifestyle behaviors such as engaging in less physical activity and more screen time, having less nocturnal sleep time and poorer dietary habits overall [7,10,11,12,13].

Breakfast consumption among children and adolescents is influenced by several socio-demographic factors including age and sex [3,4,14,15]. Other socio-demographic factors associated with higher breakfast skipping among children are single-parent homes and lower socioeconomic status, which is reflected through either family income, parental education, or occupation [1,3,14,16]. However, while a large cross-sectional study reported an inverse association between breakfast consumption and socioeconomic status in most European and North American regions, such an association with breakfast intake may reflect socio-cultural perceptions rather than socioeconomic status itself [14]. In fact, a study on school children in Pakistan reported contrasting findings, showing that children from upper socioeconomic backgrounds and higher parental education reported an increased prevalence of breakfast skipping [17].

Education plays a vital role in achieving Saudi Arabia’s Vision 2030 targets [18]. Currently, public schools in Saudi Arabia dominate K–12 education, accounting for 87% of student enrollment [19]. However, private schools in the country have increased in the last five years by 13% [19]. Future private school enrollment is expected to increase much further, as the Saudi government is targeting an increase of 25% in private school enrollment by the year 2025 [18]. The growth in the private school market is being driven by an increase in population, the government’s Vision 2030 education initiatives, and increased emphasis on parental attitudes and thinking toward the importance of their children’s education [19]. An interesting finding from a longitudinal study conducted in Britain showed that private school children reported fewer behavioral problems over time than public schoolers [20]. However, after controlling for school selection criteria, the privately-educated children appeared to be less risk averse and drink at younger ages than their public school counterparts, and the study concluded that private education added little positive value to the children’s social-emotional development [20]. Nevertheless, there are some concerns about how much private schools can offer for the overall development of child including eating habits and lifestyle behaviors [20].

Globally, it appears that there are marked socioeconomic disparities among students from public and private schools [21], which are reflected in the association between socioeconomic and health-related factors [21,22,23,24,25] or in the consumption of minimally processed and ultra-processed foods [26]. In addition, there is a scarcity of information on the differences in breakfast eating habits and lifestyle behaviors between children in public and private schools, despite the differences in the socioeconomic status of families of children attending private schools compared to those attending public schools [21]. Additionally, published studies in Saudi Arabia have been focusing on the prevalence of breakfast skipping in general and its associations with lifestyle behaviors without paying attention to the disparities in breakfast eating and lifestyle habits between public and private schools [27,28,29,30,31]. Generally speaking, research evaluating the differences in eating habits and lifestyle behaviors relative to school type is limited. Therefore, the present study aimed to assess the breakfast eating habits and lifestyle behaviors among primary school children attending public versus private schools in Jeddah, Saudi Arabia, while adjusting for socio-demographic confounders.

## 2. Materials and Methods

### 2.1. Participants

The sample was drawn from students in public and private elementary schools in the city of Jeddah, western Saudi Arabia. A multistage stratified cluster random sampling technique was utilized to select the students. The total sample size was estimated to be 960 boys and girls, based on a proportion of 0.50 with a 95% confidence level and an error of 4%. This figure included an additional 20% to the sample size to account for non-responders and missing data. The stratification scheme included gender (boys and girls schools), geographical locations of the schools (east, west, north, and south), and type of school (public versus private). Two private and four public schools were randomly selected from each area. Within each school, one class section was randomly chosen from each of the six grade levels in the elementary school. All students in the selected classes were then invited to participate in this study. The final sample size was 1149 children (523 boys, 626 girls). This represents 869 and 280 students from public and private schools, respectively. A more detailed description of the study design, sampling, and procedure has been previously published [27,28].

Ethical approval for the study was secured from the Institutional Review Board (IRB) at Princess Nourah bint Abdulrahman University, Riyadh (IRB Log Number: 19-0014). Written consent was obtained from the parents of all participating children. In addition, approval for conducting this research in schools was obtained from the Jeddah Directorate of Schools, Ministry of Education, and from the principals of all selected schools.

### 2.2. Measurements and Procedures

Body weight (to the nearest 100 g) and height (to the nearest 0.1 cm) were measured using calibrated portable medical scales (Seca 869, Birmingham, UK) with minimal clothing and without shoes. Body mass index (BMI) was calculated as the ratio of weight in kilograms divided by the squared height in meters. The extended International Obesity Task Force (IOTF) age- and sex-specific BMI cutoff reference standards were used to classify underweight, normal weight, and overweight or obesity relative to the child’s age [32]. In addition, socio-demographic measurements included age, sex, maternal and paternal age, education level, and family income.

The breakfast intake frequency as well as food choices and preferences were assessed using a specifically designed self-reported questionnaire [27,28]. Common breakfast choices that were listed in the questionnaire included: egg, cheese, peanut butter, or hummus sandwiches; pizza; ready-to-eat cereals; potato; sausage; cookies or muffins as well as local food choices. Information about the school canteen and parent satisfaction with the food provided in the school’s canteen were included. The questionnaire also contained items related to the most influencing factors on breakfast choices by Saudi children. The instrument was previously developed and content validated [27]. Questions relating to socio-demographic factors and selected lifestyle behaviors were also included in the questionnaire. The questionnaires were to be filled by the children’s parents or adult guardians.

Assessment of screen viewing time included questions on the typical daily time that the child spent on screen time including time spent viewing TV, playing video games, and on computer and Internet recreational use. This included hours spent on screen time during weekdays and weekends. Sedentary behaviors were then categorized into a cutoff based on above or below two hours per day [33].

Physical activity was evaluated using the total daily time spent by the child on all types of physical activity including sports during which the child’s breathing was increased considerably. The sufficient physical activity level was based on 60 min or more of daily physical activity [33]. Physical activity was categorized as low or high activity based on a minimal recommended cut-off value of 420 total minutes per week [33].

In addition, we assessed nocturnal sleep duration on weekdays (school days) and weekends. Parents were asked to provide how many hours their children usually slept at night on weekdays and weekends. Insufficient sleep (short sleepers) was defined as sleeping less than 9 h per night, according to the National Sleep Foundation for school-age children 6–13 years [34].

### 2.3. Statistical Analysis

Data were entered into a SPSS data file, checked, cleaned, and analyzed using IBM-SPSS program, version 22 (Chicago, IL, USA). Descriptive statistics were obtained for all variables and reported as means and standard deviations or percentages. Differences in descriptive characteristics of the participants were tested using two-way analysis of variance (school type by gender). The proportions and Chi-square tests were used to test the differences in socio-demographic and lifestyle parameters of the participants relative to school type. Multivariable analysis using two (public versus private schools) by two (boys versus girls) multivariate analysis of variance were used to test the differences in selected breakfast, BMI, and lifestyle variables while controlling for age and socio-demographic factors. Wilks’ Lambda *p*-values were reported. Moreover, logistic regression analysis while adjusting for age, gender, and socio-demographic factors was used to test the differences between children attending public versus private schools in breakfast intake, overweight or obesity, and selected lifestyle behaviors. Adjusted odds ratios (aOR) and confidence intervals (95% CI) were reported. Alpha level at 0.05 or less was set as significant.

## 3. Results

Table 1 presents the characteristics of the participants relative to gender and type of school. The mean age (SD) of the sample was 9.2 (1.7) years, ranging from 5.8 to 13 years. Two-way analysis of covariance tests (gender by school type), while controlling for age, revealed no significant differences in body weight, height, BMI, or overweight or obesity status. However, there was a significant (*p* = 0.029) gender by school interaction effect on breakfast intake (days/week). In addition, there were no differences in the proportions of daily breakfast intake between children in public (20.6%) and private (19.6%) schools. Additionally, there was no significant difference in the proportion of children having breakfast five or more days per week between public (39.8%) and private (42.1%) schools. Although there was no overall difference in the prevalence of daily breakfast intake relative to gender in public schools, boys (26.3%) in private schools showed higher (*p* = 0.006) daily breakfast intake than girls (13.3%).

The socio-demographic characteristics and lifestyle behaviors of Saudi children relative to school type are displayed in Table 2. Higher father education level (*p* < 0.001) and greater family income (*p* = 0.002) were significantly associated with private school enrollment. Moreover, two lifestyle behaviors were significantly and positively associated with private school enrollment including sleep duration at or above nine hours per night (*p* = 0.003) and high weekly physical activity (*p* = 0.042). Compared to children in public schools, children in private schools spent a higher amount of time being active at or above 420 min per week (*p* = 0.018). Table 3 shows the results of two-way MANCOVA for selected breakfast, BMI, and lifestyle variables while controlling for age and socio-demographic factors stratified by gender and school type (public versus private). There were no significant differences relative to gender, school type, or gender by school type interactions in breakfast intake, BMI, or sleep duration. However, there were significant (*p* < 0.001) gender differences in screen time and physical activity.

Table 4 shows the parent responses to questions on the school canteen stratified by school type. More than 56% of all children ate and drank from the school canteen, with significant differences (*p* < 0.001) between school types. It appears that more parents of children in private (12.1%) than in public (6.9%) schools were satisfied (*p* = 0.003) with the food offered to their children by the school canteen. Overall, more than one-third of parents were either satisfied or somewhat satisfied with the drink choices offered by the schools without any significant differences between public and private schools.

The most influencing factors on breakfast choices by Saudi children relative to school type are presented in Table 5. Children’s desire, food taste, and parental influence were chosen as the top factors that impacted on children’s breakfast choices. Surprisingly, only 16% of children were influenced by TV advertisements on their preference of breakfast choice. Table 6 shows the parents’ perceptions regarding the health quality of children’s common breakfast choices relative to school type. Egg sandwich, thyme sandwich, breakfast cereals from whole wheat or oats, yogurt, tuna sandwich, fava beans (Foul), and chickpeas (hummus) scored above 70% as healthy breakfast choices by the parents. The following breakfast choices exhibited significant (<0.050) differences in parents’ responses relative to school type: breakfast cereals, fava beans, Oreo biscuits, Nutella hazelnut spread sandwich, and hamburgers.

Finally, the results of the logistic regression analysis of selected lifestyle behaviors, adjusted for age, gender, and socio-demographic factors, relative to school type are presented in Table 7. Among all variables in the regression model, only older age (aOR = 0.889, 95% CI = 0.815–0.970, *p* = 0.008), higher father education (aOR = 1.380, 95% CI = 1.130–1.686, *p* = 0.002), higher family income (aOR = 1.227, 95% CI = 1.005–1.498, *p* = 0.044), and insufficient sleep duration (aOR = 0.740, 95% CI = 0.553–0.990, *p* = 0.042) appeared significantly associated with child enrollment in a private school.

## 4. Discussion

The current research investigated the breakfast eating habits and lifestyle behaviors among Saudi primary school children attending public versus private schools. The major findings indicated that there was low prevalence of breakfast intake among both children in the public and private schools. Additionally, a gender by school type interaction effect in breakfast intake was found, as boys in private but not in public schools showed significantly higher daily breakfast intake than girls. More parents of children in private than in public schools were satisfied with the foods provided in the school canteen and that children’s desire, food taste, and parental influence were the top factors impacting children’s breakfast choices in both types of schools. In addition, younger age, higher father education, family income, and sufficient sleep duration were significantly associated with being in a private school. No significant differences, when adjusted for socio-demographic factors, appeared in breakfast intake or overweight/obesity relative to school type.

From the findings of the present study, which showed almost 20% daily breakfast intake, and comparing this figure to the results from another cross sectional study conducted two decades ago that reported that 85% of school-age children and adolescents consumed breakfast on a daily basis [30], it appears that the trend of daily breakfast consumption in Jeddah has declined significantly. In Riyadh, another large city in Saudi Arabia, the proportion of primary school students who consumed breakfast daily had also declined from about 80% in 1999 to nearly 21% in 2017 [27,35]. Although this is in line with the declining global trend of daily breakfast consumption in most countries around the world, Saudi Arabia now ranks among those with the lowest figures of breakfast consumption: a large study using data obtained from 31 countries by a World Health Organization collaborative study on health behaviors of school-aged children found that the proportion of adolescents who consumed breakfast daily ranged between 37.8% in Slovenia to 72.6% in the Netherlands [36]. The large gap between breakfast intake prevalence in our sample and that reported for children in European and North American countries [36] points to a need to consider other countries’ policies and programs regarding breakfast intake in order to implement similar strategies in Saudi Arabia in an effort to increase daily breakfast consumption among school-aged children.

Previous research has shown that girls tend to skip breakfast more frequently than boys [1], a finding that was significant only among private school students in our sample. One explanation in previously existing literature is the girls’ concern for weight or body image [16,37]. Private school students typically come from families of higher socioeconomic status (the current findings showed that children in private schools came from parents with significantly higher income and paternal education compared with children in public schools), however, there were no significant differences between the children skipping breakfast at home relative to high income status (the percentages of children with non-daily breakfast intake in private and public schools earning more than 20,000 Saudi Riyals were 12.6% and 13.0%, respectively). Nonetheless, we did not assess (and account for) the number of children in the family, a factor that may influence enrolling children in private schools. It is also more likely that children with higher family income and education to own more personal electronic devices and have more accessibility to social media platforms, which propagate body image issues and insecurities, and thus may be skipping breakfast to counteract such insecurities [38]. Further investigation into this matter is needed to explore Saudi girls’ reasons for skipping breakfast and the associations with body image perceptions and BMI status.

In the present study, multivariable analysis did not show any significant gender differences in breakfast intake frequency. However, the boys spent significantly more time on screen viewing and engaging in physical activity than the girls. These findings were in line with earlier research showing that adolescent females in three major Saudi cities were significantly more sedentary and less physically active than their male counterparts [31]. Reliance on automotive transportation, limited physical education in schools [31], cultural acceptance of females playing sports as well as higher accessibility of males to sports clubs than females were all factors that contributed to the above-mentioned gender differences in physical activity. However, there has been a growing shift in recent years in cultural perceptions as well as in increased opportunities for young Saudi girls to actively participate in physical activity and organized sports.

After controlling for confounders (age, gender, and socioeconomic factors), children in our study who attend private schools were found to have less insufficient sleep (longer sleep duration) than their public school counterparts. While previous research has shown that longer nocturnal sleep is positively associated with breakfast consumption [10,13], we found no significant differences in daily breakfast consumption between private and public school students after adjusting for possible confounders. However, there is some evidence in the literature indicating that breakfast consumption is higher among public compared with private Indian school students [39]. Furthermore, the present study showed that higher family income correlated positively with children’s enrollment in private schools. Additionally, higher paternal but not maternal education levels were positively associated with enrollment in private schools. This finding may need future investigation in light of the fact that more Saudi females than Saudi males hold college degrees. Nevertheless, research showed that differences exist in the socioeconomic status of families of children attending private schools compared to those in public schools [15].

The current study showed that the frequency of breakfast consumption was not associated with BMI. Such findings appeared similar to those reported recently for primary school children from Riyadh [27]. The link between higher frequency of breakfast consumption and healthier BMI scores has been established in the literature [2,7,8,16,40]. Our present findings did not observe any association between the type of school and BMI status (e.g., overweight or obesity levels). An earlier study conducted on children from Riyadh, using similar methodology did find a significant interaction effect in BMI between type of school and gender [26]. Elsewhere, the prevalence of over-nutrition was significantly higher among Ethiopian primary school children attending private schools compared to those in public schools [41]. Additionally, a cross sectional investigation involving children from Ghana showed that amongst factors related to overweight or obesity was higher enrollment in private schools [22]. In contrast, a longitudinal analysis from 2005 to 2016 of overweight and obesity among Argentinian children found significant changes in adiposity in boys and girls attending public schools but not in children attending private schools [42].

Our results showed that parents of children enrolled in private schools were more satisfied with the food choices offered by the school canteen than parents of children in public schools. This could be due to better quality food and drink options sold at private school canteens and more organized food set-up. However, a recent study conducted on Saudi public high schools in Riyadh found that the actual implementation of and compliance with the guidelines on school canteen food and drink regulations published by the Ministry of Education in coordination with the Ministry of Health is limited [43]. None of the schools from the aforementioned study sold any fruits or vegetables, and instead commonly sold croissants, potato chips, fried foods, and confectionery, all of which are energy-dense nutrient-poor foods. According to the findings of an Australian study, most children perceived canteen food and drink to be ‘a treat’ and thus purposely purchased unhealthy items [44]. More importantly, we found that more than a quarter of the parents in our study were unaware of the food and drink choices sold in the canteen. This is alarming because as major stakeholders, parents can have a strong say in promoting healthy food choices in their children’s school canteen.

Breakfast choices among children in our study were mostly determined by the child’s desire, food taste, and parental influence, without significant differences in the proportions of these factors between public and private schools. These findings highly correspond with results from a large cross-sectional study on European adolescents in which the top factors were child hunger levels, food taste, and parental influence, in addition to concern for health, which was not listed as an independent choice in our survey [45]. Given our finding that the child’s desire was the most influencing factor, we can assume that Saudi children enjoy at least some level of autonomy in choosing their own foods, although the extent of such autonomy of children choosing their own breakfast items at home is yet unclear. The analysis of the dietary habits of normal-weight and obese children in Saudi Arabia also found that children who ate breakfast at home had higher odds of obesity, indicating that the breakfast quality of obese children is possibly suboptimal [46]. This leaves room for future interventions to include children in addition to parents in order to educate all family members about healthy breakfast options. However, friends as an influencing factor was found to be significantly different between public and private school attendees. Since a lower proportion of private school students bought food and drink from the canteen, this implies they are bringing more food from home, which potentially comprises a variety of unique food and drinks not otherwise found in the canteen. As such, students in private schools may be more influenced by their peers’ food and drink choices compared to students in public schools, where they generally eat a more uniform array of food all from the school canteen.

Furthermore, while only a small proportion of parents answered that TV advertisements influenced their child’s breakfast choices, research has shown that television food marketing is potentially as powerful on children as parental influence [47]. While television as the primary platform for advertising to children has declined in recent years, other media such as Internet websites are largely being used nowadays to market various foods and beverages to children [47,48]. It is worth noting that food marketing to children in Saudi Arabia is mostly unregulated, especially on online platforms. Therefore, educating parents about the magnitude of online marketing children in Saudi Arabia is needed.

Finally, while we found that most parents are generally knowledgeable about the health quality of common breakfast choices, there are still several misconceptions regarding unhealthy food choices, namely Oreo biscuits, hazelnut chocolate spread sandwiches, hot dogs, French fries, and donuts, which were rated as ‘somewhat healthy’ by a large proportion of parents. Parental knowledge, attitudes, and behaviors regarding healthy food and proper child nutrition directly and strongly influence their children’s dietary quality and eating habits [48]. Higher parental knowledge of healthy eating and active implementation of such knowledge in the home was associated with decreased child obesity risk in Riyadh [46]. This area requires in-depth exploration of Saudi parenting styles and other cultural factors involved in order to design tailored interventions to improve parental knowledge about diet quality, correct misperceptions regarding food, and educate them about appropriate child-feeding practices [48].

### 4.1. Strengths and Limitations

This study adds to the limited literature regarding breakfast eating habits and lifestyle behaviors of primary school children relative to public or private school attendance. We included a large and representative sample of Saudi children from both public and private schools. However, there are some noteworthy limitations of this study. We did not specifically measure the composition or quality of breakfast items consumed by children in this study. A major limitation in our study is the administration of surveys by the parents or guardians of children, as this leaves room for self-reporting and social-desirability biases, both of which can provide inaccuracies in results. In addition, the varying definitions of ‘breakfast consumption’ and ‘breakfast skipping’ in the literature leaves room for speculation whether eating breakfast at 9 a.m. during school recess has similar beneficial effects on primary school children as eating breakfast early in the morning before going to school. Finally, due to the large sample size, we used a subjective measure of physical activity rather than a more objective assessment such as motion sensors. This may have impacted on the accuracy of the physical activity assessment.

### 4.2. Future Implications

From the findings of the present study, it was clear that future study needs to thoroughly consider investigating the reasons that girls in private study consumed breakfast at home less frequently than girls in public schools or even lower than boys in private schools. This is occurring despite the fact that children in private schools were generally eating food from the school canteen less frequently than those in public schools. Future interventions should aim to educate parents about fostering and promoting several healthy lifestyle behaviors for their children including sufficient nocturnal sleep, daily breakfast consumption, and regular physical activity, as they are all strongly part of the healthier lifestyle behaviors in children. Furthermore, culturally-appropriate education for parents about healthy nutrition and appropriate child dietary practices is warranted. Educational campaigns and interventions on healthy breakfast food choices should aim to include both parents and children. Further research into several key areas is also needed including but not limited to children’s perceptions of weight, body image, and meal skipping, especially for girls as well as for the analysis of the dietary quality of specific food and drinks sold in school canteens.

## 5. Conclusions

The majority of children attending public and private schools do not consume breakfast daily, and girls in private schools skip breakfast significantly more than boys. Most children eat or drink from the school canteen, while most parents (more so in private schools) were somewhat satisfied with the foods provided in the school’s canteen. Children’s desire, food taste, and parental influence were the top factors influencing children’s breakfast choices in both types of schools. In addition, younger age, higher father education, family income, and sufficient sleep duration were significantly associated with being in a private school. No significant differences, when adjusted for socio-demographic factors, appeared in breakfast intake or overweight or obesity status relative to school type.

## Figures and Tables

**Table 1:** Characteristics of the participants relative to gender and type of school.

Variable	School Type	All *N* = 1149	Boys *N* = 523	Girls N = 626	*p*-Value ^1^
Age (years) (mean ± SD)	Public	9.4 ± 1.6	9.3 ± 1.6	9.5 ± 1.6	Gender: 0.785 School type: 0.198 Gender by school interactions: 0.310
	Private	9.1 ± 1.7	9.1 ± 1.7	9.0 ± 1.7	
Body weight (kg) (mean ± SD)	Public	33.0 ± 11.5	32.4 ± 11.2	33.5 ± 11.6	Gender: 0.745 School type: 0.672 Gender by school interactions: 0.289
	Private	32.5 ± 10.8	32.7 ± 10.5	32.2 ± 11.2	
Body height (cm) (mean ± SD)	Public	133.4 ± 11.9	133.3 ± 10.8	133.5 ± 12.0	Gender: 0.567 School type: 0.573 Gender by school interactions: 0.156
	Private	132.5 ± 11.0	133.5 ± 9.6	131.5 ± 12.2	
Body mass index (kg/m^2^) (mean ± SD)	Public	18.1 ± 3.9	17.8 ± 4.0	18.3 ± 3.8	Gender: 0.196 School type: 0.933 Gender by school interactions: 0.633
	Private	18.0 ± 4.0	17.9 ± 4.1	18.2 ± 4.0	
Breakfast intake (days/week) (mean ± SD)	Public	3.76 ± 2.3	3.65 ± 2.3	3.84 ± 2.3	Gender: 0.730 School type: 0.951 Gender by school interactions: 0.029
	Private	3.77 ± 2.3	4.02 ± 2.4	3.52 ± 2.1	
Daily breakfast intake (%)	Public	20.6	19.4	21.5	0.446
	Private	19.6	26.3	13.3	0.006
Breakfast intake (%) >5 days/week	Public	39.8	38.3	41.0	0.444
	Private	42.1	46.7	37.8	0.147
Overweight or obesity by school type (%)	Public	27.8	25.0	30.1	0.097
	Private	31.5	34.3	28.9	0.329

^1^ Two-way ANOVA-tests or Chi-square tests for the proportion. Data are displayed as means ± standard deviations or percentages.

**Table 2:** Socio-demographic characteristics and lifestyle behaviors of the participants relative to school type.

Variable	All	Public Schools	Private Schools	*p*-Value ^1^
**Gender (%)**				0.188
Boys	45.5	44.4	48.9	
Girls	54.5	55.6	51.1	
**Parent answering the questionnaire (%)**				0.096
Father	36.5	38.1	31.4	
Mother	60.5	58.8	66.1	
Someone else	3.0	3.1	2.5	
**Number of children in the family (%)**				0.109
1–2	15.8	15.5	16.8	
3–4	51.2	49.8	55.3	
≥5	33.0	34.6	27.9	
**Number of family members in the house (%)**				0.189
1–3	5.7	5.4	6.4	
4–5	41.8	40.5	45.7	
≥6	52.5	54.1	47.9	
**Father’s age (%)**				0.206
<30 years	0.6	0.8	0.0	
30–39 years	30.5	29.4	33.7	
40–49 years	49.1	50.5	44.9	
50–59 years	16.8	16.2	18.8	
≥60 years	3.0	3.1	2.5	
**Mother’s age (%)**				0.669
<30 years	8.5	8.2	9.6	
30–39 years	62.0	62.9	58.9	
40–49 years	26.2	25.7	27.9	
50–59 years	3.3	3.2	3.6	
≥60 years	0.00	0.00	0.00	
**Father’s education (%)**				<0.001
Intermediate or less	12.8	13.8	9.6	
High school	28.7	30.6	22.5	
University degree	49.4	48.2	53.2	
Post graduate degree	9.1	7.4	14.6	
**Mother’s education (%)**				0.357
Intermediate or less	11.1	12.0	8.6	
High school	29.4	29.7	28.6	
University degree	55.2	54.3	57.8	
Post graduate degree	4.3	4.0	5.0	
**Family income (%)** ^2^				0.002
≤10,000 SR	44.5	46.6	37.9	
10,001–20,000 SR	39.2	38.9	40.0	
20,001–30,000 SR	12.1	10.1	18.2	
≥30,001 SR	4.2	4.4	3.9	
**Breakfast intake** (daily versus non-daily)				0.730
Non-daily	79.6	79.4	80.4	
Daily	20.4	20.6	19.6	
**Breakfast intake** (5+ versus < 5 days/week)				0.490
>5 days/week	59.6	60.2	57.9	
<5 days/week	44.4	39.8	42.1	
**Screen time**				0.965
≤2 h/day	30.5	30.5	30.4	
>2 h/day	69.5	69.5	69.6	
**Sleep duration**				0.003
<9 h/night	65.8	68.1	58.6	
≥9 h/night	34.2	31.9	41.4	
**Physical activity (%)**				0.042
No physical activity	52.2	53.3	48.9	
<30 min/day	21.0	21.3	20.0	
30 min/day to <60 min/day	11.6	11.6	11.4	
60 min/day	9.2	7.9	13.3	
>60 min/day	6.0	5.9	6.4	
**Physical activity/inactivity (%)**				0.018
Low active (<420 min/week)	84.8	86.2	80.4	
High active (≥420 min/week)	15.2	13.8	19.6	
**Means of travelling to school (%)**				0.147
Walking	4.3	4.3	4.3	
Family or private car	88.0	89.0	85.4	
School bus	7.7	6.8	10.4	
**BMI category (%)** ^3^				0.233
<25 kg/m^2^	71.3	72.2	68.5	
≥25 kg/m^2^	28.7	27.8	31.5	

^1^ Chi-square tests for the differences in the proportions between public and private schools. ^2^ SR = Saudi Riyal = 3.75 USD. ^3^ Based on International Obesity Task Force (IOTF) age- and sex-specific BMI cutoff reference standards to classify normal weight and overweight or obesity [32].

**Table 3:** Multivariable analysis for selected breakfast, body mass index (BMI), and lifestyle variables while controlling for age and socio-demographic factors stratified by gender and school type (public versus private).

Variable	Gender	School Type		*p*-Value ^1^
		Public	Private	
Breakfast Intakes (day/week)	**Boys**	3.65 ± 2.3	4.02 ± 2.4	School type: 0.994 Gender: 0.624 School type by gender interactions: 0.166
	**Girls**	3.83 ± 2.3	3.54 ± 2.1	
	**All**	3.75 ± 2.3	3.77 ± 2.3	
BMI (kg/m^2^)	**Boys**	17.8 ± 4.0	18.0 ± 3.9	School type: 0.207 Gender: 0.187 School type by gender interactions: 0.759
	**Girls**	18.3 ± 3.8	18.2 ± 4.0	
	**All**	18.1 ± 3.9	18.1 ± 4.0	
Screen time (hours/night)	**Boys**	3.38 ± 1.7	3.22 ± 1.6	School type: 0.339 Gender: <0.001 School type by gender interactions: 0.894
	**Girls**	2.83 ± 1.6	2.68 ± 1.6	
	**All**	3.08 ± 1.7	2.95 ± 1.6	
Sleep duration (hours/night)	**Boys**	8.20 ± 1.2	8.64 ± 0.98	School type: 0.076 Gender: 0.238 School type by gender interactions: 0.133
	**Girls**	8.43 ± 1.2	8.44 ± 1.2	
	**All**	8.33 ± 1.2	8.54 ± 1.1	
Physical activity (minutes/week)	**Boys**	199.1 ± 229.3	214.1 ± 231.2	School type: 0.442 Gender: <0.001 School type by gender interactions: 0.543
	**Girls**	90.5 ± 159.2	106.0 ± 154.1	
	**All**	139.2 ± 201.1	159.1 ± 202.8	

Data are means and standard deviations. ^1^ Wilks’ Lambda *p*-values: age <0.001; father age = 0.036; mother age = 0.085; father’s education = 0.014; mother’s education = 0.022; family income = 0.377; school type = 0.286; gender <0.001; and school type by gender interaction = 0.509.

**Table 4:** Parent responses related to school canteen by school type.

Variable	School Type			*p*-Value ^1^
	All *N* = 1149	Public *N* = 869	Private *N* = 280	
**Does your child eat or drink from the school’s canteen?**				<0.001
Eats & drinks from the school canteen	56.3	59.4	46.8	
Eats only from the school canteen	21.7	22.0	20.7	
Does not eat or drink from the school canteen	15.6	12.8	24.3	
Drinks only from the school canteen	6.4	5.9	8.2	
**Are parents satisfied with the foods provided in the school’s canteen?**				0.003
Yes, satisfied	8.2	6.9	12.1	
Somewhat satisfied	32.6	33.6	32.6	
Not satisfied	31.9	33.5	26.8	
Does not know about the foods in the school canteen	27.4	26.0	31.8	
**Are parents satisfied with the drinks provided in the school’s canteen?**				0.090
Yes, satisfied	14.8	13.8	17.9	
Somewhat satisfied	31.9	33.4	27.5	
Not satisfied	25.8	26.4	23.9	
Does not know about the drinks in the school canteen	27.5	26.5	30.7	

^1^ Chi-square tests for significant differences in the proportions between public and private schools.

**Table 5:** Factors influencing breakfast choices by Saudi children relative to school type (more than one choice was possible).

Variable	School Type			*p*-Value ^1^
	All	Public	Private	
Child’s wish or desire	55.4	55.9	53.9	0.559
Foods taste	39.9	41.2	35.7	0.103
Parental influence	33.9	33.8	34.3	0.889
Brothers or sisters	17.9	17.5	19.3	0.496
Child’s friends	17.4	15.7	22.9	0.006
TV promotions (TV ads)	16.1	16.9	13.6	0.185
Other factors	1.1	1.3	0.4	0.193

^1^ Chi-square tests for significant differences in the proportions between public and private schools.

**Table 6:** Chi-square tests for significant differences in the proportions between public and private schools.

Variable	School Type	Healthy	Somewhat Healthy	Not Healthy	*p*-Value ^1^
Egg sandwich	Public	91.4	7.4	1.3	0.742
	Private	92.1	7.1	0.7	
Thyme sandwich	Public	82.7	15.8	1.5	0.334
	Private	81.4	15.7	2.9	
Breakfast cereals from whole wheat or oats	Public	81.5	17.3	1.2	0.047
	Private	81.8	15.0	3.2	
Yogurt with fruits	Public	79.1	17.4	3.6	0.802
	Private	77.5	18.2	4.3	
Tuna sandwich	Public	75.0	22.4	2.5	0.185
	Private	80.4	17.9	1.8	
Fava beans (Foul)	Public	67.3	28.4	4.3	0.050
	Private	75.0	22.1	2.9	
Chickpeas (Hummus)	Public	69.3	26.7	4.0	0.152
	Private	73.9	21.1	5.0	
Peanut butter sandwich	Public	44.5	45.3	10.1	0.083
	Private	51.4	37.9	10.7	
Solid cheese sandwich	Public	30.5	53.6	15.9	0.694
	Private	33.2	51.4	15.4	
Pancake	Public	19.8	55.1	25.1	0.495
	Private	21.8	51.1	27.1	
Croissant	Public	15.1	54.3	30.6	0.683
	Private	13.2	56.8	30.0	
Spread cheese sandwich	Public	11.1	36.0	52.9	0.896
	Private	11.1	37.5	51.4	
Pizza	Public	9.7	49.1	41.2	0.906
	Private	8.9	50.4	40.7	
Mortadella sandwich (cured meat)	Public	6.0	34.2	59.8	0.101
	Private	7.5	27.5	65.0	
Oreo biscuit	Public	5.9	37.3	56.8	0.024
	Private	10.4	32.5	57.1	
Donuts	Public	4.9	32.9	62.1	0.170
	Private	7.1	28.2	64.6	
Nutella sandwich (hazelnut chocolate spread)	Public	4.3	32.8	62.9	0.050
	Private	7.9	29.6	62.5	
French fries	Public	2.9	25.0	72.1	0.317
	Private	4.3	27.5	68.2	
Hamburger	Public	2.3	26.2	71.5	0.002
	Private	6.4	27.9	65.7	
Hot dog	Public	2.9	22.4	74.7	0.676
	Private	3.2	20.0	76.8	

^1^ Chi-square tests for significant differences in the proportions between public and private schools.

**Table 7:** Results of logistic regression analysis of selected lifestyle behaviors, adjusted for gender, and socio-demographic factors, relative to school type among Saudi children.

Variable	Public versus Private School ^1^			
	aOR	(95% CI)	SEE	*p*-Value
Age (younger age = ref)	1.00			
Older age	0.889	0.815–0.970	0.044	0.008
Gender (girls = ref)	1.00			
Boys	0.868	0.649–1.161	0.148	0.341
Father age (older age = ref)	1.00			
Younger age	1.129	0.911–1.398	0.109	0.268
Mother age (older age = ref)	1.00			
younger age	1.071	0.825–1.391	0.133	0.606
Father education (low = ref)	1.00			
High education	1.380	1.130–1.686	0.102	0.002
Mother education (low = ref)	1.00			
High education	0.958	0.773–1.186	0.109	0.693
Family income (low = ref)	1.00			
High income	1.227	1.005–1.498	0.102	0.044
Screen time (high = ref)	1.00			
Low screen time	1.336	0.995–1.794	0.158	0.868
Sleep duration (sufficient = ref)	1.00			
Insufficient sleep	0.740	0.553–0.990	0.149	0.042
Physical activity (high active = ref)	1.00			
Low active	0.756	0.518–1.102	0.193	0.146
Breakfast intake frequency (non-daily = ref)	1.00			
Daily	1.131	0.795–1.608	0.180	0.495
Overweight or obesity (overweight/obesity = ref)	1.00			
Non-overweight/non-obesity	0.816	0.603–1.106	0.155	0.190

^1^ Public school was used as a reference category. aOR = adjusted odds ratio; CI = confidence interval; ref = reference category; SEE = standard error.

## Data Availability

All data generated or analyzed during this study are included in this published article. Any additional data are available from the corresponding author on reasonable request.
